# Clinical application of common inflammatory and nutritional indicators before treatment in prognosis evaluation of non-small cell lung cancer: a retrospective real-world study

**DOI:** 10.3389/fmed.2023.1183886

**Published:** 2023-07-13

**Authors:** Xiang Lv, Bin Xu, Qingxia Zou, Songtao Han, Yangchun Feng

**Affiliations:** ^1^Clinical Laboratory Center, Cancer Hospital Affiliated to Xinjiang Medical University, Ürümqi, China; ^2^Department of Laboratory Medicine, Jianyang People's Hospital, Jianyang, China; ^3^Medical Laboratory Center, Affiliated to Traditional Chinese Medicine Hospital of Xinjiang Medical University, Ürümqi, China

**Keywords:** NSCLC, prognosis, big data, inflammation, immunoglobulin

## Abstract

**Objective:**

To evaluate the prognostic value of common clinical inflammatory and nutritional indicators before treatment in patients with non-small cell lung cancer in the real world.

**Method:**

A total of 5,239 patients with pathologically confirmed non-small cell lung cancer from 2011 to 2018 in the Affiliated Cancer Hospital of Xinjiang Medical University were selected. Their inflammatory and nutritional indicators (RDW, PDW, NLR, LMR, NMR, PLR, SII, PNI, TP, ALB, CYRFA21-1, CEA, CA125, NSE, α1-globulin, α2-globulin, β1-globulin, β2-globulin, and γ-globulin) before treatment were collected. From the total number, 1,049 patients were randomly sampled (18 to 20% of patients each year) and used as the validation set; the remaining 4,190 patients were used as the training set. According to the eighth edition of the guidelines for the diagnosis, treatment, and stage risk stratification of lung cancer, the patients were divided into four groups: stage I/II operable, stage III operable, stage III inoperable, and stage IV. We used the X-tile software to intercept and classify the cut-off values of each index in the validation set. Univariate and multivariate Cox proportional-hazard regression were used to screen the independent risk factors affecting the prognosis of non-small cell lung cancer and establish a prognostic model for 1, 3, and 5 years. The validation set was used to verify its performance. Finally, the Kaplan–Meier curve was used to assess the survival rate, and the corresponding nomogram was established for clinical use.

**Results:**

After screening, no effective indicators were found in the stage I/II operable group. RDW and CA125 were effective indicators for the stage III operable group (cut-off values were 14.1 and 9.21, respectively, compared with the low-value group; univariate HR was 2.145 and 1.612, and multivariate HR was 1.491 and 1.691, respectively). CYRFA21-1 and CA125 were effective prognostic indicators for the stage III inoperable group (cut-off values were 10.62 and 44.10, respectively, compared with the low-value group; univariate HR was 1.744 and 1.342, and multivariate HR was 1.284 and 1.304, respectively). CYRFA21-1, CA125, NLR, and α1-globulin were effective indicators of prognosis in stage IV (cut-off values were 3.07, 69.60, 4.08, and 5.30, respectively, compared with the low-value group; univariate HR was 1.713, 1.339, 1.388, and 1.539; and multivariate HR was 1.407, 1.119, 1.191, and 1.110, respectively). The model was constructed with the best validation power in stage IV patients (C-index = 0.733, 0.749, and 0.75 at 1, 3, and 5 years, respectively).

**Conclusion:**

For patients with stage III and IV non-small cell lung cancer, some inflammatory markers, serum tumor markers, and nutritional indicators are independent prognostic factors. Combined with the general data of patients, the constructed prognostic evaluation model has the best efficacy in patients with stage IV and can be widely used in clinical practice.

## 1. Background

Lung cancer ranks second in all cancer incidence and first in cancer mortality worldwide. Age-standardized morbidity and mortality rates for lung cancer are 22.4 and 18.0 per 100,000, respectively. Lung cancer incidence and mortality are associated with factors such as the Human Development Index (HDI), gross domestic product (GDP), and smoking frequency (1). Based on pathological types, lung cancer can be broadly categorized into small cell lung cancer (SCLC) and non-small cell lung cancer (NSCLC), of which NSCLC is mainly squamous cell carcinoma and adenocarcinoma; adenocarcinoma occupies 30–40% of lung cancer (2). Since 2010, with advances in diagnosis and surgery, such as standardized division of pathological stages, improvement in thoracoscopic surgery, drug therapy for driver mutations, and application of immune checkpoint inhibitors, the survival rate of patients with non-small cell lung cancer has significantly improved (3–8).

At present, with the study of the tumor microenvironment, more and more studies suggest that inflammation plays an essential role in the occurrence, development, and prognosis of malignant tumors (9, 10). In particular, NLR, LMR, NMR, PLR, SII, and other inflammatory indicators have been confirmed to affect the prognosis of various malignant tumors (11–13). Since malignant tumors are consumptive diseases, their nutritional indicators, such as Alb and PNI, are also important factors affecting various types of tumors (14, 15). Classical serum NSCLC tumor markers such as CA125, CYFRA21-1, and CEA are also thought to influence the prognosis of malignant tumors (16, 17).

Given the importance of inflammation and nutrition in cancer progression and prognosis, peripheral blood leukocyte-related detection indicators and nutrition-related biochemical indicators are routine detection items for clinical patients, and enough data are easily obtained. However, the prognostic value of inflammatory and nutritional indicators in real-world cases of big data in clinical healthcare facilities has rarely been reported. In this study, we combined and continuously investigated the prognostic impact of inflammation, nutritional indicators, and protein components on different stages of NSCLC, providing a brand-new idea.

## 2. Method

### 2.1. Patient selection

We retrospectively selected 5,239 patients with pathologically diagnosed NSCLC, including lung squamous cell carcinoma and adenocarcinoma, from 2011 to 2018 in the Affiliated Cancer Hospital of Xinjiang Medical University and collected their pretreatment hematological parameters. A total of 9,824 cases of lung cancer diagnosed over the past 10 years were included, including 286 cases of metastatic lung cancer, 3,845 cases of primary small cell lung cancer, 5,693 cases of non-small cell lung cancer, 335 cases where driver gene positivity was excluded, and 119 cases of unknown treatment. Finally, 5,239 cases were selected, which included 549 cases of stage I, 213 cases of stage II, 1,417 cases of stage III, and 3,060 cases of stage IV NSCLC. Among them, a total of 1,049 patients, approximately 18 to 20% of patients each year, were randomly sampled as the validation set, and the rest were used as the training set. All were approved by the ethics committee of the tumor hospital affiliated with Xinjiang Medical University.

#### 2.1.1. Inclusion criteria

(1) Primary NSCLC with definite pathology; (2) complete hematological parameters can be collected; (3) complete follow-up time and outcome; and (4) clear treatment plan.

#### 2.1.2. Exclusion criteria

(1) Pre-operative radiotherapy and chemotherapy; (2) less than one cycle of chemotherapy and radiotherapy; (3) death within 30 days after treatment; (4) history of systemic inflammation associated with active infection; and (5) gene-driven positivity or use of gene-driven positivity drugs (mainly in stage IV patients).

All patients underwent pretreatment assessments, including medical history, treatment regimen, pathologic diagnosis, routine hematology and serum immunology tests, chest radiography, electrocardiography, chest and upper abdominal CT, brain magnetic resonance imaging (MRI), and bronchoscopy. Whole body bone scan or positron emission tomography-computed tomography (PET-CT) was performed when metastasis was suspected.

### 2.2. Demographic and clinical variables

#### 2.2.1. Demographic

(1) Patients in stage I/II disease underwent radical surgery, including complete resection of the primary tumor (lobectomy), mediastinal lymph node dissection, and minimally invasive radical resection of lung cancer. Some patients underwent postoperative adjuvant chemotherapy, including platinum doublet adjuvant chemotherapy, with chemotherapy cycles >1 cycle; (2) Patients in stage III disease underwent surgery, and individualized radiotherapy and chemotherapy, with more than one cycle. Hematological parameters were collected within 1 week before surgery. (3) Patients in stages III and IV who did not undergo surgery underwent individualized radiotherapy and chemotherapy with more than one cycle. Hematological parameters were collected within 1 week before treatment.

#### 2.2.2. Indicators

Collection indicators included validation indicators, complete blood cell count, neutrophil to lymphocyte ratio (NLR), neutrophil to monocyte ratio (NMR), lymphocyte to monocyte ratio (LMR), platelet to lymphocyte ratio (PLR), red blood cell distribution width (RDW), platelet distribution width (PDW), and SII inflammatory indicator calculated by platelet count (g/L) × neutrophil count (10^9^/L)/lymphocyte count (10^9^/L). Nutritional indicators included total protein (TP), albumin (Alb), and PNI nutritional index collected by albumin (g/L) + 5x lymphocyte count (10^9^/L). Tumor marker results collected included glycoprotein-125 (CA125), carcinoembryonic antigen (CEA), a soluble fragment of cytokeratin 19 (CYRFA21-1), neuron-specific enolase (NSE), and protein electrophoresis results. Demographic baseline and clinicopathological characteristics, including age, sex, family history, cancer treatment, history of lung-related diseases, pathological type, and TNM stage, were obtained from medical records.

### 2.3. Follow up

After their first follow-up stage, patients were followed up every 3 months for 1 year, every 6 months for 2 to 3 years, and then once a year. These follow-ups included hematological parameters, CT, MRI, PET-CT, etc. The last follow-up was in January 2022, and overall survival (OS) was calculated from the date of diagnosis to the date of death/last follow-up, with an average OS time of 23.9 months.

### 2.4. Statistics

Quantitative data were described using means ± standard deviations, analysis of variance was used to compare quantitative data, and the Chi-square test or Fisher's exact test was used to compare categorical variables. Using the training set data, X-tile software was used to determine the optimal cut-off value for quantitative data and divided into two categories, and all categorical data were described using percentages. Univariate and multivariate Cox proportional-hazard regression was performed using SPSS software to screen independent risk factors for prognosis, and nomogram prognostic models were established using R software. Calibration and time-dependent ROC curves were validated, training set data were used for internal validation, and finally, the Kaplan-Meier method was used to draw survival curves. The log-rank test was used to assess survival differences. All *p* values < 0.05 were considered significant.

## 3. Results

### 3.1. Clinical and demographic characteristics

A total of 5,239 NSCLC patients were included in the study, with a mean age of 61.54 years, including 4,190 patients in the training set and 1,049 patients in the validation set. As seen in Table 1, in the training and validation sets, males (*n* = 2,599, 634) and adenocarcinomas (*n* = 2,783, 746) accounted for the majority. Among the TNM stage, pT1 (*n* = 375, 100) and pT2 (*n* = 379, 81) accounted for the majority of patients with stage I, II, and III operable early stage. Most of them underwent postoperative adjuvant chemotherapy (*n* = 454, 95), while among patients with stage III un-operated and stage IV advanced stage, T3 (*n* = 565, 138) and T4 (*n* = 1,187, 303) accounted for the majority. In the training and validation sets, there were more patients receiving chemotherapy treatment (*n* = 2,299, 577) than those receiving radiotherapy treatment (*n* = 617, 155). Among the included indicators, except for nutritional indicators PNI, TP, and ALB, which tended to decrease with stage, the other indicators tended to increase. In the difference analysis, except in the following, the indicators were significant in different stage groups (*p* < 0.05): in the training set, PDW (*p* = 0.131), NLR (*p* = 0.133), LMR (*p* = 0.079), NMR (*p* = 0.150), and β1-globulin (*p* = 0.370); in the validation set, gender (*p* = 0.089), pathological type (*p* = 0.216), RDW (*p* = 0.939), PDW (*p* = 0.732), LMR (*p* = 0.277), NMR (*p* = 0.090), SII (*p* = 0.184), and β1-globulin (*p* = 0.516).

**Table 1:** Clinical and demographic characteristics.

**Variables**	**Training set**					**Validation set**				
	**Stage I/II operable**	**Stage III operable**	**Stage III inoperable**	**Stage IV**	**P**	**Stage I/II operable**	**Stage III operable**	**Stage III inoperable**	**Stage IV**	***P***
**Gender**					0.002					0.089
Male	347 (57.3%)	180 (58.4%)	621 (74.3%)	1,451 (59.5%)		85 (54.5%)	37 (57.8%)	159 (76.1%)	353 (56.9%)	
Female	259 (42.7%)	128 (41.6%)	215 (25.7%)	989 (40.5%)		71 (45.5%)	27 (42.2%)	50 (23.9%)	267 (43.1%)	
**Age**	61.36 ± 10.00	58.72 ± 10.40	63.20 ± 10.32	61.38 ± 11.30	< 0.001	61.71 ± 10.24	58.28 ± 10.58	62.68 ± 9.38	61.64 ± 11.28	0.039
**Pathological type**					< 0.001					0.939
Adenocarcinoma	169 (27.9%)	292 (62.3%)	387 (46.3%)	1,935 (79.3%)		119 (76.3%)	37 (57.8%)	95 (45.5%)	495 (79.8%)	
Squamous cell carcinoma	437 (72.1%)	116 (37.7%)	449 (53.7%)	505 (20.7%)		37 (23.7%)	27 (42.2%)	114 (54.5%)	125 (20.2%)	
**T**					< 0.001					< 0.001
(p)T1 (a/b)	310 (51.2%)	65 (21.1%)	73 (8.7%)	285 (11.7%)		88 (56.4%)	12 (18.8%)	19 (9.1%)	72 (11.6%)	
(p)T2 (a/b)	249 (41.1%)	130 (42.2%)	264 (31.6%)	902 (37.0%)		62 (39.7%)	19 (29.7%)	63 (30.3%)	234 (37.7%)	
(p)T3	47 (7.7%)	59 (19.2%)	202 (24.2%)	363 (14.9%)		6 (3.8%)	18 (28.1%)	48 (23.0%)	90 (14.5%)	
(p)T4	0	54 (17.5%)	297 (35.5%)	890 (36.5%)		0	15 (23.4%)	79 (37.8%)	224 (36.1%)	
**N**					< 0.001					< 0.001
(p)N0	507 (83.7%)	19 (6.2%)	31 (3.7%)	233 (9.5%)		138 (88.5%)	4 (6.3%)	7 (3.3%)	59 (9.5%)	
(p)N1	99 (16.3%)	28 (9.1%)	59 (7.1%)	190 (7.8%)		18 (11.5%)	10 (15.6%)	16 (7.7%)	52 (8.4%)	
(p)N2	-	244 (79.2%)	443 (53.0%)	1,038 (42.5%)		0	45 (70.3%)	121 (57.9%)	259 (41.8%)	
(p)N3	-	17 (5.5%)	303 (36.2%)	979 (40.1%)		0	5 (7.8%)	65 (31.1%)	250 (40.3%)	
**M**					< 0.001					< 0.001
M0	606 (100%)	308 (100%)	836 (100%)	0		156 (100%)	64 (100%)	209 (100%)	0	
M1	-	-	-	2,440 (100%)		0	0	0	620 (100%)	
**Stage**					-					-
Ia/b	426 (70.1%)	-	-	-		123 (78.8%)	-	-	-	
IIa/b	180 (29.7%)	-	-	-		33 (21.2%)	-	-	-	
IIIa	-	228 (77.2%)	263 (31.0%)	-		-	42 (65.6%)	77 (36.8%)	-	
IIIb	-	59 (20.0%)	435 (51.2%)	-		-	20 (31.2%)	95 (45.5%)	-	
IIIc	-	8 (2.8%)	151 (17.8%)	-		-	2 (3.2%)	37 (17.7%)	-	
IV	-	-	-	2,440 (100%)		-		-	620 (100%)	
**Chemotherapy**					< 0.001					< 0.001
Yes	216 (35.6%)	238 (77.3%)	569 (68.1%)	1,730 (70.9%)		52 (33.3%)	43 (67.2%)	150 (71.8%)	427 (68.9%)	
No	390 (64.4%)	70 (22.7%)	267 (31.95%)	710 (29.1%)		104 (66.7%)	21 (32.8%)	59 (28.2%)	193 (31.1%)	
**Radiation therapy**					< 0.001					< 0.001
Yes	0	79 (25.6%)	230 (27.5%)	387 (15.9%)		0	16 (25.0%)	64 (30.6%)	91 (14.7%)	
No	606 (100%)	227 (74.4%)	606 (72.5%)	2,053 (84.1%)		156 (100%)	48 (75.0%)	145 (69.4%)	529 (85.3%)	
**RDW**	13.11 ± 1.25	13.26 ± 1.16	13.41 ± 1.57	13.52 ± 1.64	< 0.001	13.20 ± 1.39	13.44 ± 1.37	13.46 ± 1.76	13.49 ± 1.58	0.216
**PDW**	11.66 ± 2.35	11.68 ± 2.65	11.54 ± 2.63	11.44 ± 2.52	0.131	11.65 ± 2.27	11.56 ± 1.98	11.57 ± 3.18	11.42 ± 2.45	0.732
**NLR**	3.98 ± 3.96	4.92 ± 14.24	4.57 ± 5.36	4.99 ± 11.27	0.133	3.82 ± 3.32	5.21 ± 5.04	4.15 ± 3.95	4.80 ± 5.19	0.042
**LMR**	5.78 ± 13.30	6.69 ± 15.30	9.37 ± 34.04	8.41 ± 30.18	0.079	4.96 ± 8.13	6.60 ± 13.30	11.55 ± 32.46	8.83 ± 40.34	0.277
**NMR**	22.10 ± 91.27	20.64 ± 45.04	26.90 ± 62.91	27.82 ± 69.77	0.150	17.90 ± 74.50	25.43 ± 54.27	36.33 ± 101.04	25.87 ± 56.33	0.090
**PLR**	174.92 ± 97.07	192.32 ± 132.82	227.19 ± 886.08	211.00 ± 363.91	< 0.001	174.77 ± 83.32	241.10 ± 227.66	203.69 ± 247.52	193.08 ± 119.85	0.033
**SII**	987.95 ± 953.88	1,087.94 ± 1,143.47	1,226.99 ± 2,282.20	1,276.53 ± 1,601.75	0.001	971.42 ± 842.67	1,322.81 ± 1,230.73	1,145.10 ± 1,545.34	1,203.63 ± 1,345.26	0.184
**PNI**	50.38 ± 12.82	48.78 ± 6.02	47.20 ± 6.94	46.20 ± 6.86	< 0.001	50.31 ± 5.18	47.31 ± 7.20	47.55 ± 6.26	46.42 ± 6.91	< 0.001
**TP**	70.11 ± 6.26	70.10 ± 6.36	70.44 ± 6.60	68.90 ± 7.05	< 0.001	69.96 ± 5.63	70.78 ± 6.64	70.70 ± 6.78	68.82 ± 7.01	0.001
**ALB**	41.85 ± 4.66	40.98 ± 4.88	39.40 ± 5.30	38.58 ± 5.50	< 0.001	42.27 ± 4.15	40.11 ± 5.36	39.61 ± 5.15	38.66 ± 5.61	< 0.001
**CYRFA21-1**	3.61 ± 8.17	6.33 ± 10.38	11.15 ± 19.83	13.32 ± 27.52	< 0.001	3.26 ± 5.47	8.42 ± 14.47	9.97 ± 15.50	12.24 ± 26.31	< 0.001
**CEA**	20.55 ± 127.54	15.68 ± 44.07	48.42 ± 170.56	97.68 ± 257.95	< 0.001	15.72 ± 96.22	26.51 ± 65.39	30.85 ± 115.22	98.74 ± 258.40	< 0.001
**CA125**	29.77 ± 81.95	41.38 ± 94.41	81.29 ± 272.26	134.62 ± 229.69	< 0.001	25.00 ± 57.41	73.49 ± 176.59	64.41 ± 142.90	134.63 ± 225.41	< 0.001
**NSE**	14.65 ± 6.58	17.42 ± 16.65	19.61 ± 21.76	3.17 ± 13.02	< 0.001	15.08 ± 8.81	15.68 ± 4.84	19.25 ± 17.75	23.85 ± 29.86	< 0.001
**α1-globulin**	4.16 ± 1.29	4.64 ± 1.87	5.35 ± 2.02	5.56 ± 1.87	< 0.001	4.11 ± 1.16	5.26 ± 2.83	5.40 ± 2.16	5.53 ± 1.87	< 0.001
**α2-globulin**	9.56 ± 2.09	10.25 ± 2.39	11.31 ± 2.67	11.84 ± 2.72	< 0.001	9.38 ± 2.07	10.66 ± 2.50	11.26 ± 2.56	11.82 ± 2.82	< 0.001
**β1-globulin**	6.06 ± 0.88	6.17 ± 1.07	6.09 ± 0.97	6.12 ± 0.99	0.370	5.99 ± 0.74	6.15 ± 0.99	6.09 ± 0.93	6.10 ± 0.92	0.516
**β2-globulin**	4.94 ± 1.18	5.18 ± 2.06	5.38 ± 1.10	5.47 ± 1.16	< 0.001	4.98 ± 1.24	5.14 ± 1.25	5.39 ± 1.10	5.50 ± 1.17	< 0.001
**γ-globulin**	17.41 ± 3.40	17.42 ± 3.43	18.51 ± 4.01	18.53 ± 3.86	< 0.001	17.19 ± 3.18	18.59 ± 4.04	18.31 ± 4.22	18.47 ± 3.98	0.003

### 3.2. Optimal cut-off values and clinical

In recent years, X-tile software has been the primary tool used to intercept optimal cut-off values. The training set was used to intercept optimal cut-off values of quantitative data of each stage and classify it. For example, the cut-off value of NLR in stage I/II operable group was 1.95. The patients were divided into two groups ( ≤ 1.95 and >1.95). Using this method, the cut-off values were intercepted for each indicator in different stage groups and expressed in Table 2. All categorical variables are expressed as percentages through analysis of clinical and demographic characteristics (Table 1).

**Table 2:** Based on the training set, the optimal cut-off value was truncated using the x-tile software and expressed as a percentage.

**Variables**	**Training set**						
	**Stage I/II operable**		**Stage III operable**		**Stage III inoperable**		**Stage IV**
**Gender**							
Male	347 (57.3%)	Male	180 (58.4%)	Male	621 (74.3%)	Male	1,451 (59.5%)
Female	259 (42.7%)	Female	128 (41.6%)	Female	215 (25.7%)	Female	989 (40.5%)
**Age**							
≤ 60	278 (45.9%)	≤ 60	167 (54.2%)	≤ 60	321 (38.4%)	≤ 60	1,094 (44.8%)
>60	328 (54.1%)	>60	141 (45.8%)	>60	515 (61.6%)	>60	1,346 (55.2%)
**Pathological type**							
Squamous cell carcinoma	169 (27.9%)	Squamous cell carcinoma	116 (37.7%)	Squamous cell carcinoma	449 (53.7%)	Squamous cell carcinoma	505 (20.7%)
Adenocarcinoma	437 (72.1%)	Adenocarcinoma	292 (62.3%)	Adenocarcinoma	387 (46.3%)	Adenocarcinoma	1,935 (79.3%)
**T**							
pT1	310 (51.2%)	p T1	65 (21.1%)	T1	73 (8.7%)	T1	285 (11.7%)
pT2	249 (41.1%)	pT2	130 (42.2%)	T2	264 (31.6%)	T2	902 (37.0%)
pT3	47 (7.7%)	pT3	59 (19.2%)	T3	202 (24.2%)	T3	363 (14.9%)
pT4	0 (0%)	pT4	54 (17.5%)	T4	297 (35.5%)	T4	890 (36.5%)
**N**							
pN0	507 (83.7%)	pN0	19 (6.2%)	N0	31 (3.7%)	N0	233 (9.5%)
pN1	99 (16.3%)	pN1	28 (9.1%)	N1	59 (7.1%)	N1	190 (7.8%)
pN2	0	pN2	244 (79.2%)	N2	443 (53.0%)	N2	1,038 (42.5%)
pN3	0	pN3	17 (5.5%)	N3	303 (36.2%)	N3	979 (40.1%)
**M**							
M0	606 (0%)	M0	308 (100.0%)	M0	836 (100%)	M0	0 (0)
M1	0	M1	0 (0)	M1	0	M1	2,440 (100%)
**Stage**							
Ia/b	426 (70.1%)	Ia/b	-	Ia/b	-	Ia/b	-
IIa/b	180 (29.7%)	IIa/b	-	IIa/b	-	IIa/b	-
IIIa	-	IIIa	228 (77.2%)	IIIa	263 (31.0%)	IIIa	-
IIIb	-	IIIb	59 (20.0%)	IIIb	435 (51.2%)	IIIb	-
IIIc	-	IIIc	8 (2.8%)	IIIc	151 (17.8%)	IIIc	-
IV	-	IV	-	IV	-	IV	2,440 (100%)
**Chemotherapy**							
Yes	216 (35.6%)	Yes	238 (77.3%)	Yes	569 (68.1%)	Yes	1,730 (70.9%)
No	390 (64.4%)	No	70 (22.7%)	No	267 (31.9%)	No	710 (29.1%)
**Radiation therapy**							
Yes	40 (6.6%)	Yes	79 (25.6%)	Yes	230 (27.5%)	Yes	387 (15.9%)
No	566 (93.4%)	No	227 (74.4%)	No	606 (72.5%)	No	2,053 (84.1%)
**RDW**							
≤ 13.11	355 (58.6%)	≤ 14.10	258 (83.8%)	≤ 13.30	470 (56.2%)	≤ 14.00	1,833 (75.1%)
>13.11	251 (41.4%)	>14.10	50 (16.2%)	>13.30	366 (43.8%)	>14.00	607 (24.9%)
**PDW**							
≤ 9.60	82 (13.5%)	≤ 9.40	34 (11.0%)	≤ 11.70	498 (59.6%)	≤ 12.70	1,883 (77.2%)
>9.60	524 (86.5%)	>9.40	274 (89.0%)	>11.70	338 (40.4%)	>12.70	557 (22.8%)
**NLR**							
≤ 1.95	136 (22.4%)	≤ 3.01	141 (45.8%)	≤ 5.31	644 (77.0%)	≤ 4.08	1,459 (59.8%)
>1.95	470 (77.6%)	>3.01	167 (54.2%)	>5.31	192 (23.0%)	>4.08	981 (40.2%)
**LMR**							
≤ 3.47	346 (57.1%)	≤ 3.22	159 (51.6%)	≤ 2.60	272 (32.5%)	≤ 2.68	966 (39.6%)
>3.47	260 (42.9%)	>3.22	149 (48.4%)	>2.60	564 (67.5%)	>2.68	1,474 (60.4%)
**NMR**							
≤ 14.64	522 (86.1%)	≤ 6.82	56 (18.2%)	≤ 11.59	490 (58.6%)	≤ 48.26	2,163 (88.6%)
>14.64	84 (13.9%)	>6.82	252 (81.8%)	>11.59	346 (41.4%)	>48.26	277 (11.4%)
**PLR**							
≤ 154.91	296 (48.8%)	≤ 304.38	274 (89.0%)	≤ 145.89	330 (39.5%)	≤ 24.92	2,067 (84.7%)
>154.91	310 (51.2%)	>304.38	34 (11.0%)	>145.89	506 (60.5%)	>24.92	373 (15.3%)
**SII**							
≤ 385.04	124 (20.5%)	≤ 892.78	168 (54.5%)	≤ 1,841.49	716 (85.6%)	≤ 1,583.10	1,879 (77.0%)
>385.04	482 (79.5%)	< 892.78	140 (45.5%)	>1,841.49	120 (14.4%)	>1,583.10	561 (23.0%)
**PNI**							
≤ 43.33	71 (11.7%)	≤ 46.95	111 (36.0%)	≤ 40.09	121 (14.5%)	≤ 43.77	859 (35.2%)
>43.33	535 (88.3%)	>46.95	197 (64.0%)	>40.09	715 (85.5%)	>43.77	1,581 (64.8%)
**TP**							
≤ 63.20	74 (12.2%)	≤ 64.40	57 (18.5%)	≤ 66.80	228 (27.3%)	≤ 60.60	605 (12.5%)
>63.20	532 (87.8%)	>64.40	251 (81.5%)	>66.80	608 (72.7%)	>60.60	2,135 (87.5%)
**ALB**							
≤ 36.10	61 (10.1%)	≤ 37.70	65 (21.1%)	≤ 35.10	172 (20.6%)	≤ 37.70	880 (36.1%)
>36.10	545 (89.9%)	>37.70	243 (78.9%)	>35.10	664 (79.4%)	>37.70	1,560 (63.9%)
**CYFRA21-1**							
≤ 3.73	491 (81.0%)	≤ 2.70	162 (52.6%)	≤ 10.62	633 (75.7%)	≤ 3.07	731 (30.0%)
>3.73	115 (19.0%)	>2.70	146 (47.4%)	>10.62	203 (24.3%)	>3.07	1,709 (70.0%)
**CEA**							
≤ 7.49	518 (85.5%)	≤ 5.56	194 (63.0%)	≤ 21.98	664 (79.4%)	≤ 15.70	1,425 (58.4%)
>7.49	88 (14.5%)	>5.56	114 (37.0%)	>21.98	172 (20.6%)	>15.70	1,015 (41.6%)
**CA125**							
≤ 42.10	539 (88.9%)	≤ 9.21	52 (16.9%)	≤ 44.10	543 (65.0%)	≤ 69.60	1,447 (59.3%)
>42.10	67 (11.1%)	>9.21	256 (83.1%)	>44.10	293 (35.0%)	>69.60	993 (40.7%)
**NSE**							
≤ 18.44	525 (86.6%)	≤ 20.06	259 (84.1%)	≤ 25.54	732 (87.6%)	≤ 27.0.71	2,026 (83.0%)
>18.44	81 (13.4%)	>20.06	49 (15.9%)	>25.54	104 (12.4%)	>27.71	414 (17.0%)
>3.90	264 (43.6%)	>4.70	99 (32.1%)	>5.50	294 (35.2%)	>5.30	1,110 (45.5%)
α**2-globulin**							
≤ 10.00	405 (66.8%)	≤ 13.30	277 (89.9%)	≤ 13.20	655 (78.3%)	≤ 11.60	1,200 (49.2%)
>10.00	201 (33.2%)	>13.30	31 (10.1%)	>13.20	181 (21.7%)	>11.60	1,240 (50.8%)
β**1-globulin**							
≤ 5.70	202 (33.3%)	≤ 5.20	39 (12.7%)	≤ 5.80	321 (38.4%)	≤ 5.50	619 (25.4%)
>5.70	404 (66.7%)	>5.20	269 (87.3%)	>5.80	515 (61.6%)	>5.50	1,821 (74.6%)
β**2-globulin**							
≤ 5.30	406 (67.0%)	≤ 5.40	223 (72.4%)	≤ 6.40	704 (84.2%)	≤ 6.10	1,910 (78.3%)
>5.30	200 (33.0%)	>5.40	85 (27.6%)	>6.40	132 (15.8%)	>6.10	530 (21.7%)
γ**-globulin**							
≤ 19.30	467 (77.1%)	≤ 21.10	269 (87.3%)	≤ 17.30	343 (41.0%)	≤ 19.70	1,546 (63.4%)
>19.30	139 (22.9%)	>21.10	391 (2.7%)	>17.30	493 (59.0%)	>19.70	894 (36.6%)

### 3.3. Univariate and multifactor Cox proportional-hazard regression analysis

According to the classification results of X-tile stages, univariate Cox proportional-hazard regression analysis was performed using the general data, pathological characteristics, and classification results of the training set (Table 2). It revealed that age, gender, depth of invasion (T), stage, pathological type, RDW, NLR, LMR, SII, PNI, TP, Alb, CYFR421-1, CEA, CA125, NSE, α1-globulin, and α2-globulin were risk factors in stage I operable group. Age, gender, T, lymph node metastasis(N), stage, RDW, PDW, NLR, LMR, NMR, PLR, SII, PNI, TP, Alb, CYFR421-1, CA125, NSE, α1-globulin, α2-globulin, β2-globulin, and γ-globulin were risk factors in stage III operable group. In the stage III inoperable group, age, gender, T, stage, pathological type, chemotherapy, radiotherapy, RDW, PDW, NLR, LMR, PLR, SII, PNI, Alb, CYFR421-1, CA125, NSE, α1-globulin, α2-globulin, β2-globulin, and γ-globulin were risk factors. Age, gender, T, N, chemotherapy, radiotherapy, RDW, PDW, NLR, LMR, PLR, SII, PNI, TP, Alb, CYFR421-1, CA125, α1-globulin, α2-globulin, β1-globulin, and γ-globulin were risk factors in the stage IV group. Based on the univariate Cox proportional-hazard regression results, multivariate Cox proportional-hazard regression analysis was performed (Table 3). It showed that age and T were independent risk factors in the stage I/II operable group. Gender, age, N, RDW, and CA125 were independent risk factors in the stage III operable group. Gender, age, stage, radiotherapy, chemotherapy, CYFRA21-1, and CA125 were independent risk factors in the stage III inoperable group. Gender, age, T, N, chemotherapy, radiotherapy, NLR, CYFRA21-1, CA125, and α1-globulin were independent risk factors in the stage IV group.

**Table 3:** Based on the training set, univariate and multivariate Cox regression analysis.

**Training set**																			
**Variables**	**Stage I/II operable**				**Variables**	**Stage III operable**				**Variables**	**Stage III inoperable**				**Variables**	**Stage IV**			
	**Univariate analysis**		**Multivariate analysis**			**Univariate analysis**		**Multivariate analysis**			**Univariate analysis**		**Multivariate analysis**			**Univariate analysis**		**Multivariate analysis**	
	**HR (95% CI)**	***p***	**HR (95% CI)**	***p***		**HR (95% CI)**	***p***	**HR (95% CI)**	***p***		**HR (95% CI)**	***p***	**HR (95% CI)**	***p***		**HR (95% CI)**	***p***	**HR (95% CI)**	***P***
**Gender**		< 0.001		0.082	**Gender**		0.001		0.024	**Gender**		< 0.001		< 0.001	**Gender**		< 0.001		< 0.001
Male	1.000		1.000		Male	1.000		1.000		Male	1.000		1.000		Male	1.000		1.000	
Female	0.543 (0.404 0.728)		0.744 (0.533 1.038)		Female	0.585 (0.429 0.798)		0.670 (0.473 0.949)		Female	0.597 (0.498 0.716)		0.675 (0.554 0.823)		Female	0.710 (0.652 0.774)		0.756 (0.693 0.826)	
**Age**		< 0.001		0.006	**Age**		0.001		0.027	**Age**		< 0.001		< 0.001	**Age**		< 0.001		0.003
< 60	1.000		1.000		< 60	1.000		1.000		< 60	1.000		1.000		< 60	1.000		1.000	
≥60	1.719 (1.295 2.280)		1.513 (1.129 2.027)		≥60	1.672 (1.246 2.244)		1.450 (1.043 2.017)		≥60	1.469 (1.256 1.719)		1.370 (1.159 1.621)		≥60	1.220 (1.121 1.327)		1.141 (1.047 1.244)	
**Pathological type**		0.037		0.246	**Pathological type**		0.095			**Pathological type**		0.006		0.090	**Pathological type**		0.075		
Squamous cell carcinoma	1.000		1.000		Squamous cell carcinoma	1.000				Squamous cell carcinoma	1.000		1.000		Squamous cell carcinoma	1.000			
Adenocarcinoma	0.738 (0.554 0.982)		1.232 (0.866 1.753)		Adenocarcinoma	0.775 (0.574 1.046)				Adenocarcinoma	0.810 (0.697 0.942)		0.863 (0.728 1.023)		Adenocarcinoma	0.621 (0.560 0.688)			
**T**		< 0.001		0.025	**T**		0.001		0.153	**T**		< 0.001		0.550	**T**		< 0.001		0.006
pT1a/b	1.000		1.000		pT1a/b	1.000		1.000		T1a/b	1.000		1.000		T1	1.000		1.000	
pT2a/b	1.523 (1.138 2.038)	0.005	1.338 (0.975 1.836)	0.071	pT2a/b	1.457 (0.934 2.272)	0.097	1.192 (0.744 1.911)	0.465	T2a/b	1.235 (0.922 1.655)	0.157	1.138 (0.845 1.532)		T2	1.291 (1.115 1.495)	0.001	1.130 (0.973 1.312)	0.110
pT3	2.911 (1.908 4.441)	< 0.001	2.149 (1.224 3.772)	0.008	pT3	2.148 (1.316 3.505)	0.002	1.427 (0.654 3.115)	0.372	p3	1.688 (1.236 2.253)	0.001	1.012 (0.731 1.401)		T3	1.531 (1.288 1.821)	< 0.001	1.129 (0.945 1.349)	0.180
pT4					pT4	2.351 (1.449 3.815)	0.001	2.119 (1.037 4.331)	0.039	T4	1.664 (1.246 2.221)	0.001	1.141 (0.838 1.554)		T4	1.569 (1.356 1.816)	< 0.001	1.274 (1.097 1.479)	0.002
**N**		0.624		0.016	**N**		0.017		0.042	**N**		0.717			**N**		< 0.001		< 0.001
pN0	1.000				pN0	1.000		1.000		N0	1.000				N0	1.000		1.000	
pN1	1.092 (0.768 1.551)				pN1	1.045 (0.467 2.337)	0.915	1.095 (0.444 2.701)	0.844	N1	1.200 (0.735 1.960)	0.466			N1	1.167 (0.945 1.441)	0.151	1.123 (0.906 1.391)	0.291
PN2					pN2	1.186 (0.624 2.256)	0.603	1.979 (0.768 5.099)	0.157	N2	1.245 (0.824 1.882)	0.298			N2	1.440 (1.232 1.683)	< 0.001	1.263 (1.075 1.483)	0.005
PN3					pN3	2.718 (1.217 6.074)	0.015	3.953 (1.331 11.741)	0.013	N3	1.274 (0.838 1.937)	0.257			N3	1.637 (1.399 1.915)	< 0.001	1.388 (1.179 1.636)	< 0.001
**Stage**		0.002		0.737	**Stage**		< 0.001		0.375	**Stage**		< 0.001		0.005	**Stage**				
Ia/b	1.000		1.000		Ia/b					Ia/b					Ia/b				
IIa/b	1.564 (1.184 2.066)		1.062 (0.748 1.508)		IIa/b					IIa/b					IIa/b				
IIIa					IIIa	1.000		1.000		IIIa	1.000		1.000		IIIa				
IIIb					IIIb	2.580 (1.871 3.559)	< 0.001	1.320 (0.715 2.436)	0.375	IIIb	1.182 (0.998 1.400)	0.053	1.280 (1.064 1.540)		IIIb				
IIIc					IIIc	2.809 (1.306 6.044)	< 0.001	1.320 (0.715 2.436)	0.375	IIIc	1.585 (1.277 1.968)	< 0.001	1.487 (1.151 1.920)		IIIc				
IV					IV					IV					IV				
**Chemotherapy**		0.201			**Chemotherapy**		0.377			**Chemotherapy**		< 0.001		< 0.001	**Chemotherapy**		< 0.001		< 0.001
No	1.000				No	1.000				No	1.000		1.000		No	1.000		1.000	
Yes	1.198 (0.908 1.579)				Yes	0.858 (0.610 1.206)				Yes	0.612 (0.523 0.716)	0.507	0.690 (0.594 0.816)		Yes	0.527 (0.480 0.578)		0.567 (0.514 0.624)	
**Radiation therapy**					**Radiation therapy**		0.492			**Radiation therapy**		< 0.001		< 0.001	**Radiation therapy**		< 0.001		< 0.001
No					No	1.000				No	1.000		1.000		No	1.000		1.000	
Yes					Yes	0.886 (0.626 1.252)				Yes	0.718 (0.606 0.850)		0.729 (0.612 0.868)		Yes	0.753 (0.670 0.847)		0.804 (0.714 0.906)	0.682
**RDW**		0.043		0.444	**RDW**		< 0.001		0.050	**RDW**		< 0.001		0.175	**RDW**		< 0.001		0.003
≤ 13.11	1.000		1.000		≤ 14.10	1.000		1.000		≤ 13.30	1.000		1.000		≤ 14.00	1.000		1.000	
>13.11	1.323 (1.009 1.733)		1.117 (0.842 1.483)		>14.10	2.145 (1.514 3.040)		1.491 (1.000 2.223)		>13.30	1.359 (1.170 1.578)		1.117 (0.952 1.311)		>14.00	1.342 (1.218 1.478)		1.163 (1.052 1.287)	
**PDW**		0.297			**PDW**		0.005		0.097	**PDW**		< 0.001		0.110	**PDW**		< 0.001		0.069
≤ 9.60	1.000				≤ 9.40	1.000		1.000		≤ 11.70	1.000		1.000		≤ 12.70	1.000		1.000	
>9.60	0.819 (0.562 1.193)				>9.40	0.543 (0.355 0.830)		0.659 (0.402 1.079)		>11.70	0.723 (0.620 0.843)		0.874 (0.740 1.031)		>12.70	0.794 (0.717 0.878)		0.907 (0.817 1.008)	
**NLR**		< 0.001		0.069	**NLR**		0.029		0.691	**NLR**		< 0.001		0.217	**NLR**		< 0.001		0.003
≤ 1.95	1.000		1.000		≤ 3.01	1.000		1.000		≤ 5.31	1.000		1.000		≤ 4.08	1.000		1.000	
>1.95	2.192 (1.463 3.284)		1.695 (0.961 2.991)		>3.01	1.391 (1.034 1.873)		1.105 (0.676 1.807)		>5.31	1.408 (1.182 1.678)		1.164 (0.915 1.482)		>4.08	1.388 (1.274 1.512)		1.191 (1.063 1.334)	
**LMR**		0.005		0.644	**LMR**		0.006		0.711	**LMR**		< 0.001		0.627	**LMR**		< 0.001		0.838
≤ 3.47	1.000		1.000		≤ 3.22	1.000		1.000		≤ 2.60	1.000		1.000		≤ 2.68	1.000		1.000	
>3.47	0.671 (0.506 0.889)		0.930 (0.683 1.266)		>3.22	0.659 (0.489 0.887)		0.942 (0.607 1.406)		>2.60	0.714 (0.610 0.835)		0.958 (0.805 1.140)		>2.68	0.708 (0.650 0.771)		0.990 (0.897 1.092)	
**NMR**		0.168			**NMR**		0.017		0.180	**NMR**		0.188			**NMR**		0.400		
≤ 13.11	1.000				≤ 6.82	1.000		1.000		≤ 11.59	1.000				≤ 48.26	1.000			
>13.11	1.288 (0.899 1.846)				>6.82	0.653 (0.461 0.927)		0.751 (0.494 1.141)		>11.59	1.106 (0.952 1.286)				>48.26	0.940 (0.830 1.077)			
**PLR**		0.146			**PLR**		0.003		0.159	**PLR**		< 0.001		0.312	**PLR**		< 0.001		0.056
≤ 154.90	1.000				≤ 304.38	1.000		1.000		≤ 145.89	1.000		1.000		≤ 312.34	1.000		1.000	
>154.90	1.222 (0.932 1.602)				>304.38	1.858 (1.233 2.801)		1.448 (0.865 2.422)		>145.89	1.364 (1.170 1.590)		1.094 (0.919 1.303)		>312.34	1.484 (1.320 1.688)		1.189 (0.995 1.420)	
**SII**		0.010		0.934	**SII**		0.010		0.772	**SII**		< 0.001		0.224	**SII**		< 0.001		0.154
≤ 385.00	1.000		1.000		≤ 892.78	1.000		1.000		≤ 1,891.49	1.000		1.000		≤ 1,583.10	1.000		1.000	
>385.00	1.655 (1.131 2.424)		1.022 (0.606 1.725)		>892.78	1.471 (1.097 1.973)		1.073 (0.665 1.734)		>1,891.49	1.576 (1.282 1.937)		1.186 (0.901 1.560)		>1,583.10	1.396 (1.264 1.542)		0.886 (0.750 1.046)	
**PNI**		< 0.001		0.996	**PNI**		0.009		0.560	**PNI**		< 0.001		0.495	**PNI**		< 0.001		0.119
≤ 13.11	1.000		1.000		≤ 46.95	1.000		1.000		≤ 40.09	1.000		1.000		≤ 43.77	1.000		1.000	
>13.11	0.465 (0.327 0.661)		1.002 (0.547 1.835)		>46.95	0.671 (0.497 0.904)		1.126 (0.755 1.678)		>40.09	0.539 (0.440 0.662)		0.899 (0.663 1.220)		>43.77	0.653 (0.598 0.713)		0.899 (0.786 1.028)	
**TP**		0.013		0.148	**TP**		0.090		0.116	**TP**		0.314			**TP**		< 0.001		0.945
≤ 63.20	1.000		1.000		≤ 64.4	1.000		1.000		≤ 66.80	1.000				≤ 60.60	1.000		1.000	
>63.20	0.627 (0.434 0.905)		0.727 (0.472 1.120)		>64.4	0.736 (0.517 1.049)		0.714 (0.470 1.086)		>66.80	0.917 (0.776 1.085)				>60.60	0.786 (0.692 0.892)		1.005 (0.871 1.159)	
**ALB**		< 0.001		0.271	**ALB**		0.007		0.953	**ALB**		< 0.001		0.615	**ALB**		< 0.001		0.123
≤ 36.10	1.000		1.000		≤ 37.7	1.000		1.000		≤ 35.1	1.000		1.000		≤ 37.70	1.000		1.000	
>36.10	0.415 (0.290 0.595)		0.707 (0.381 1.311)		>37.7	0.627 (0.445 0.882)		1.015 (0.613 1.681)		>35.1	0.579 (0.484 0.693)		0.932 (0.709 1.226)		>37.70	0.648 (0.594 0.707)		0.901 (0.789 1.029)	
**CYFR421-1**		< 0.001		0.144	**CYFR421 1**		< 0.001		0.167	**CYFR421 1**		< 0.001		0.009	**CYFR421 1**		< 0.001		< 0.001
≤ 3.73	1.000		1.000		≤ 2.70	1.000		1.000		≤ 10.62	1.000		1.000		≤ 3.07	1.000		1.000	
>3.73	2.332 (1.664 2.995)		1.319 (0.910 1.912)		>2.70	1.744 (1.299 2.343)		1.277 (0.903 1.805)		>10.62	1.744 (1.471 2.067)		1.284 (1.065 1.549)		>3.07	1.713 (1.559 1.883)		1.407 (1.272 1.556)	
**CEA**		< 0.001		0.115	**CEA**		0.188			**CEA**		0.674			**CEA**		0.162		
≤ 7.49	1.000		1.000		≤ 5.56	1.000				≤ 21.98	1.000				≤ 15.70	1.000			
>7.49	1.832 (1.314 2.555)		1.353 (0.929 1.971)		>5.56	1.225 (0.905 1.658)				>21.98	0.962 (0.801 1.154)				>15.70	0.941 (0.864 1.025)			
**CA125**		< 0.001		0.143	**CA125**		0.025		0.022	**CA125**		< 0.001		0.002	**CA125**		< 0.001		0.015
≤ 42.10	1.000		1.000		≤ 9.21	1.000		1.000		≤ 44.10	1.000		1.000		≤ 69.60	1.000		1.000	
>42.10	2.191 (1.535 3.127)		1.386 (0.899 2.083)		>9.21	1.612 (1.062 2.447)		1.691 (1.079 2.651)		>44.10	1.342 (1.149 1.567)		1.304 (1.102 1.543)		>69.60	1.339 (1.229 1.458)		1.119 (1.022 1.226)	
**NSE**		0.027		0.990	**NSE**		0.035		0.167	**NSE**		0.013		0.968	**NSE**		0.088		
≤ 18.44	1.000		1.000		≤ 20.06	1.000		1.000		≤ 25.54	1.000		1.000		≤ 27.71	1.000			
>18.44	1.486 (1.046 2.112)		0.998 (0.679 1.466)		>20.06	1.506 (1.031 2.199)		1.354 (0.881 2.080)		>25.54	1.325 (1.062 1.654)		0.995 (0.784 1.264)		>27.71	1.713 (1.535 1.911)			
**α1-globulin**		0.003		0.566	**α1 globulin**		0.005		0.918	**α1 globulin**		< 0.001		0.103	**α1 globulin**		< 0.001		0.048
≤ 3.90	1.000		1.000		≤ 4.70	1.000		1.000		≤ 5.50	1.000		1.000		≤ 5.30	1.000		1.000	
>3.90	1.513 (1.155 1.981)		1.100 (0.794 1.525)		>4.70	1.545 (1.137 2.099)		0.978 (0.646 1.482)		>5.50	1.804 (1.545 2.105)		1.199 (0.964 1.491)		>5.30	1.539 (1.414 1.675)		1.110 (1.001 1.232)	
**α2-globulin**		0.007		0.953	**α2 globulin**		0.004		0.897	**α2 globulin**		< 0.001		0.234	**α2 globulin**		< 0.001		0.089
≤ 3.90	1.000		1.000		≤ 13.30	1.000		1.000		≤ 13.20	1.000		1.000		≤ 11.60	1.000		1.000	
>3.90	1.461 (1.109 1.925)		1.010 (0.732 1.393)		>13.30	1.929 (1.232 3.019)		0.962 (0.535 1.730)		>13.20	1.853 (1.553 2.210)		1.159 (0.909 1.477)		>11.60	1.534 (1.410 1.670)		1.094 (0.986 1.213)	
**β1-globulin**		0.062			**β1 globulin**		0.343			**β1 globulin**		0.089			**β1 globulin**		0.002		0.157
≤ 5.70	1.000				≤ 5.20	1.000				≤ 5.80	1.000				≤ 5.50	1.000		1.000	
>5.70	1.443 (1.067 1.953)				>5.20	0.812 (0.528 1.249)				>5.80	0.826 (0.709 0.961)				>5.50	0.862 (0.783 0.949)		0.930 (0.842 1.028)	
**β2-globulin**		0.079			**β2 globulin**		0.002		0.121	**β2 globulin**		0.020		0.770	**β2 globulin**		0.153		
≤ 5.30	1.000				≤ 5.40	1.000		1.000		≤ 6.40	1.000		1.000		≤ 6.10	1.000			
>5.30	0.778 (0.588 1.030)				>5.40	1.638 (1.195 2.244)		1.342 (0.925 1.945)		>6.40	1.275 (1.040 1.563)		0.968 (0.778 1.204)		>6.10	1.313 (1.187 1.453)			
**γ-globulin**		0.088			**γ** **globulin**		0.073			**γ** **globulin**		0.001		0.198	**γ** **globulin**		< 0.001		0.886
≤ 19.30	1.000				≤ 21.10	1.000				≤ 17.30	1.000		1.000		≤ 19.70	1.000		1.000	
>19.30	1.687 (1.259 2.261)				>21.10	1.457 (0.966 2.196)				>17.30	1.283 (1.102 1.494)		1.110 (0.947 1.301)		>19.70	1.224 (1.122 1.335)		0.993 (0.905 1.090)	

### 3.4. Establish a nomogram prognostic model

**Figure 1:**
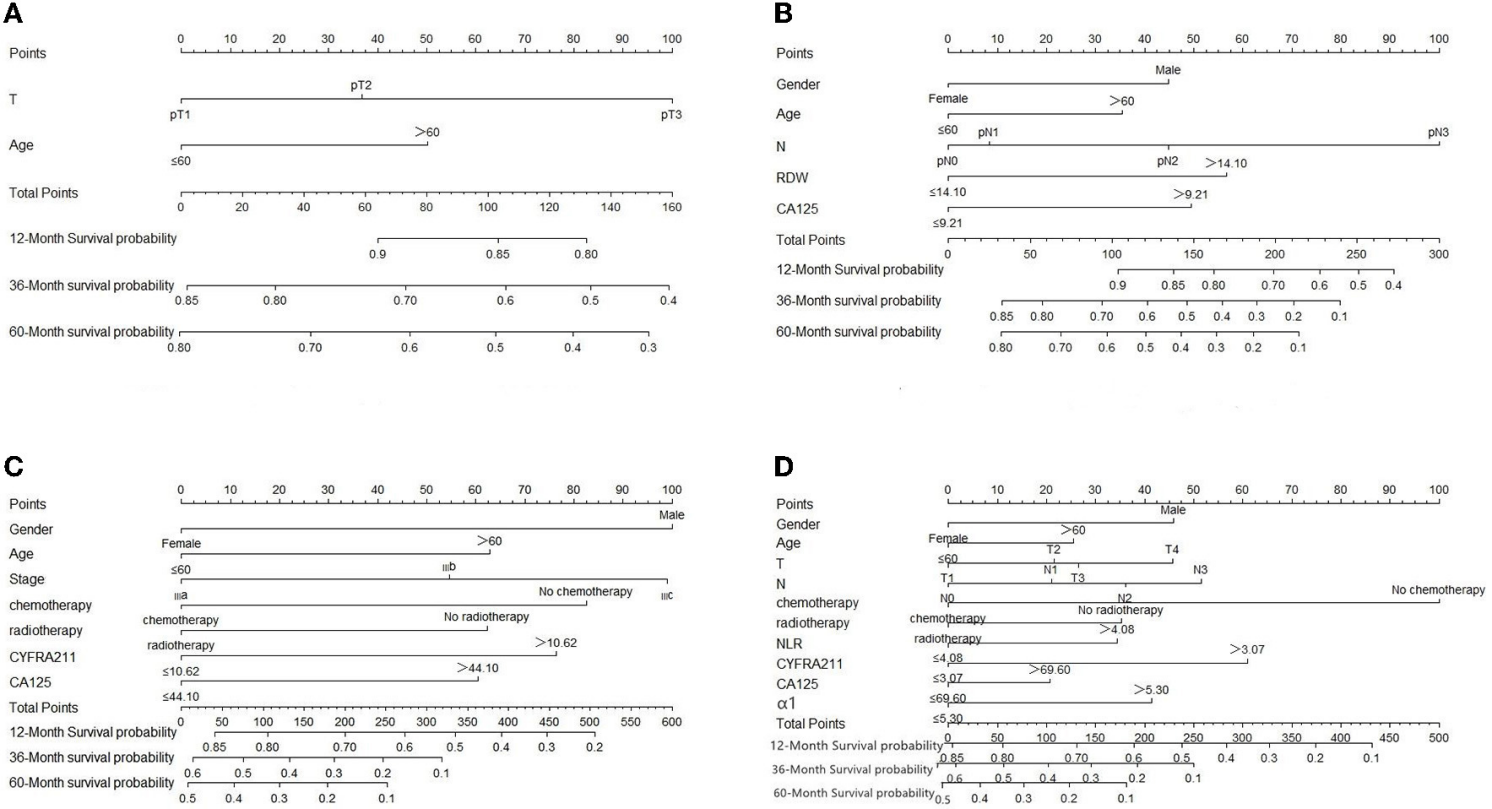
**(A–D)** Evaluation of NSCLC nomograms by stage. To use nomograms, there is a patient value on each variable axis, and a line is drawn up to determine the number of points for each variable value. The sum of these numbers lies on the total score axis, and a line is drawn down to the survival axis to determine the likelihood of survival at 1, 3, and 5 years.

A nomogram prognostic model (Figures 1A–D) was established based on a multivariate Cox proportional-hazard regression analysis of the training set. Each value level of each factor was scored according to the degree of contribution of each factor to the outcome variable in the model (the magnitude of the regression coefficient). Then each score was summed to obtain the total score. Finally, the predictive value of the individual outcome event was calculated by the functional transformation relationship between the total score and the probability of the outcome event.

### 3.5. Prognostic model performance validation

**Figure 2:**
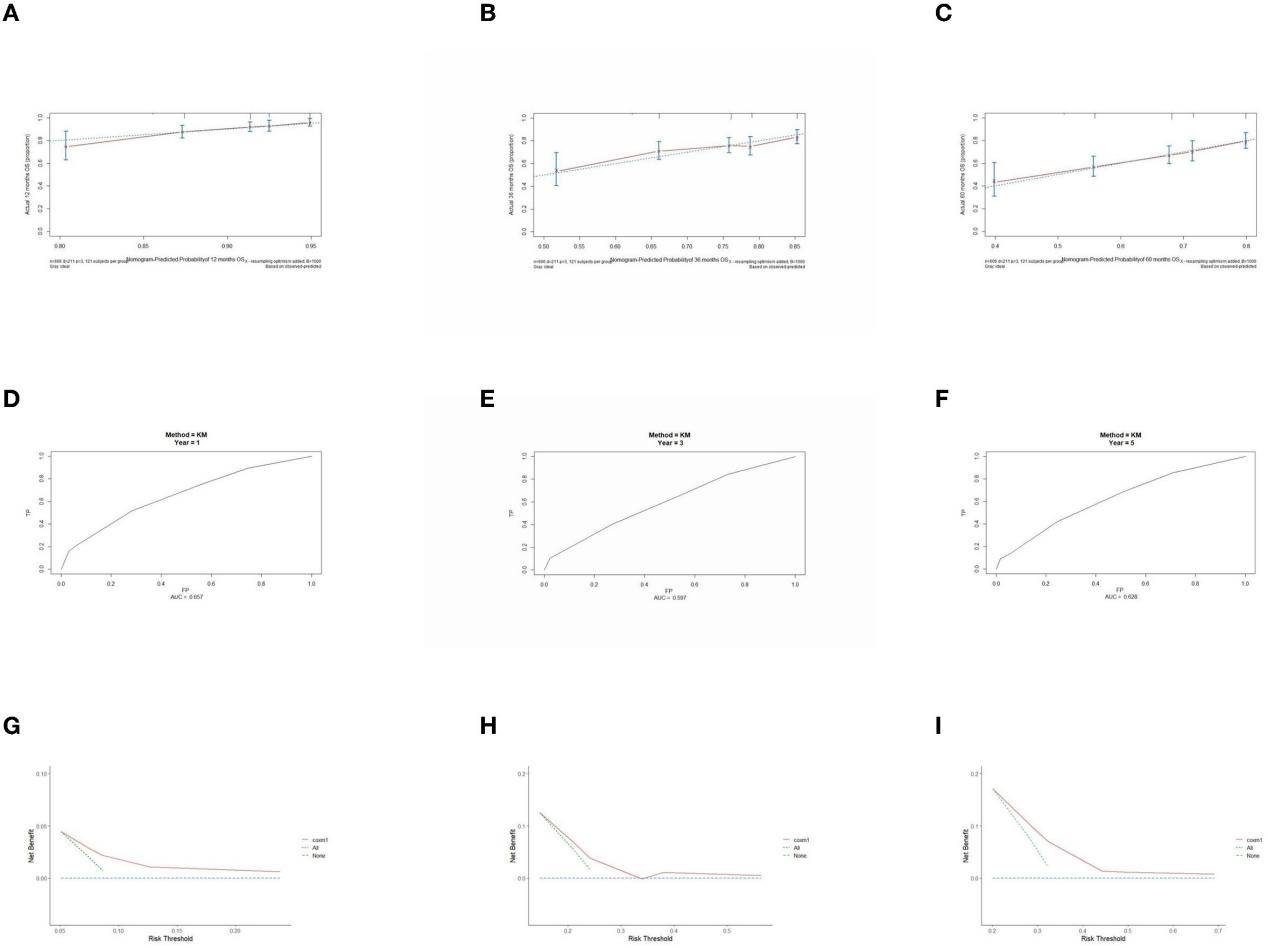
Performance verification of 1,3 and 5 year nomograms for stage I/II operable in the training set. Nomograms 1,3 and 5 year calibration curves **(A–C)**, 1,3 and 5 year time dependent ROC curves **(D–F)**, 1,3 and 5 year decision curve analysis **(G–I)**.

**Figure 3:**
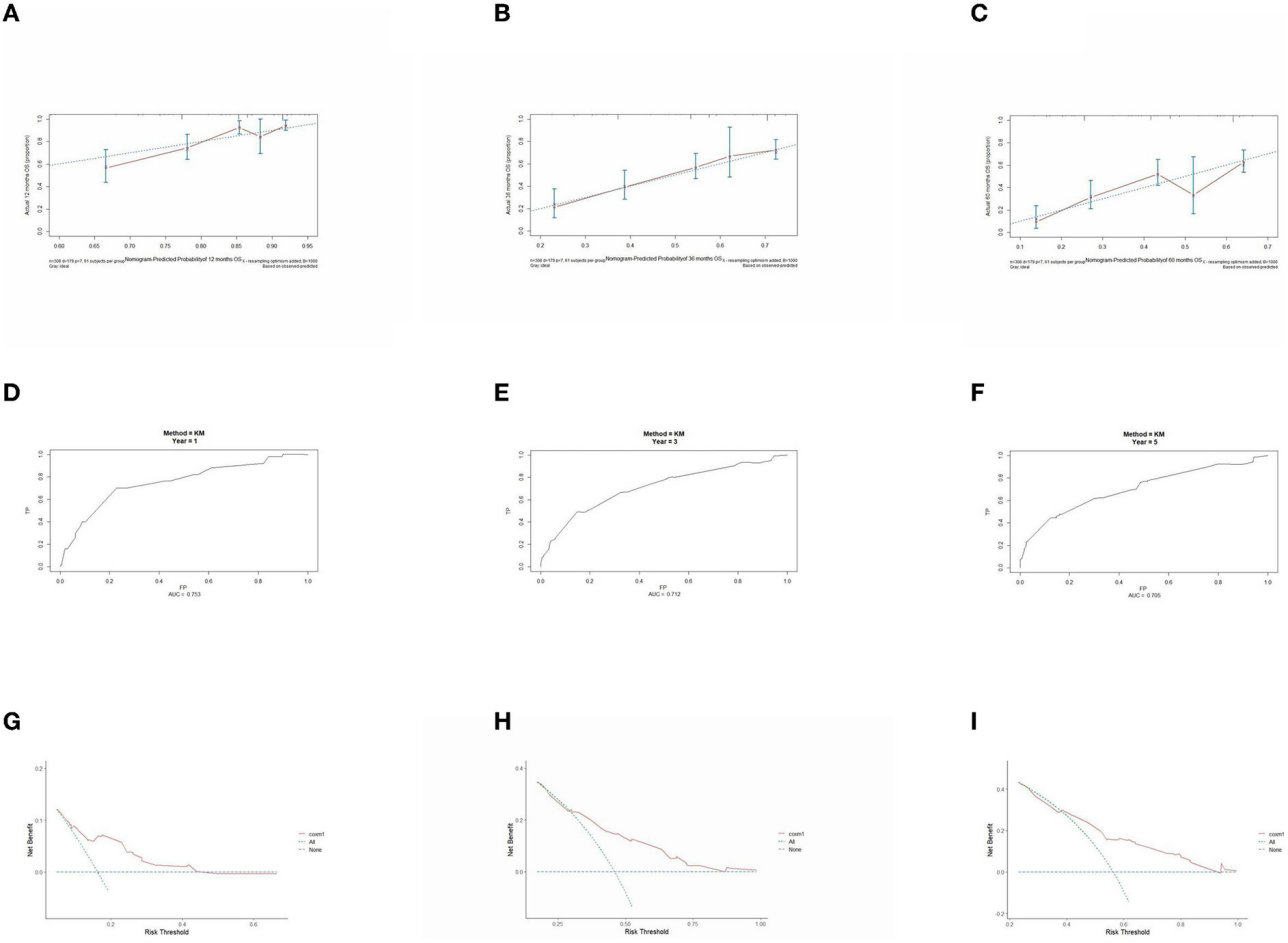
Performance verification of 1,3 and 5 year nomograms for stage III operable in the training set. Nomograms 1,3 and 5 year calibration curves **(A–C)**, 1,3 and 5 year time dependent ROC curves **(D–F)**, 1,3 and 5 year decision curve analysis **(G–I)**.

**Figure 4:**
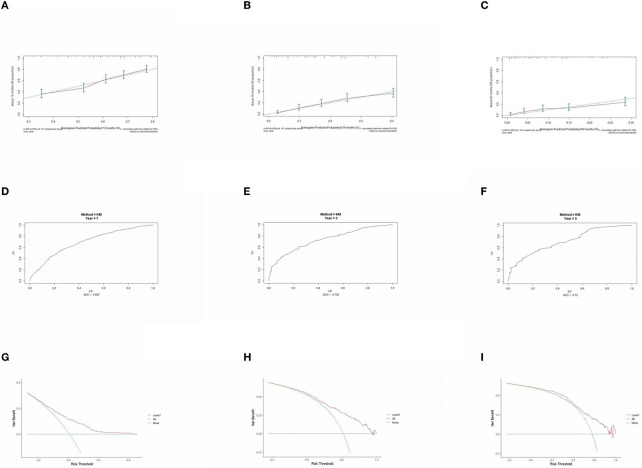
Performance verification of 1,3 and 5 year nomograms for stage III inoperable in the training set. Nomograms 1,3 and 5 year calibration curves **(A–C)**, 1,3 and 5 year time dependent ROC curves **(D–F)**, 1,3 and 5 year decision curve analysis **(G–I)**.

**Figure 5:**
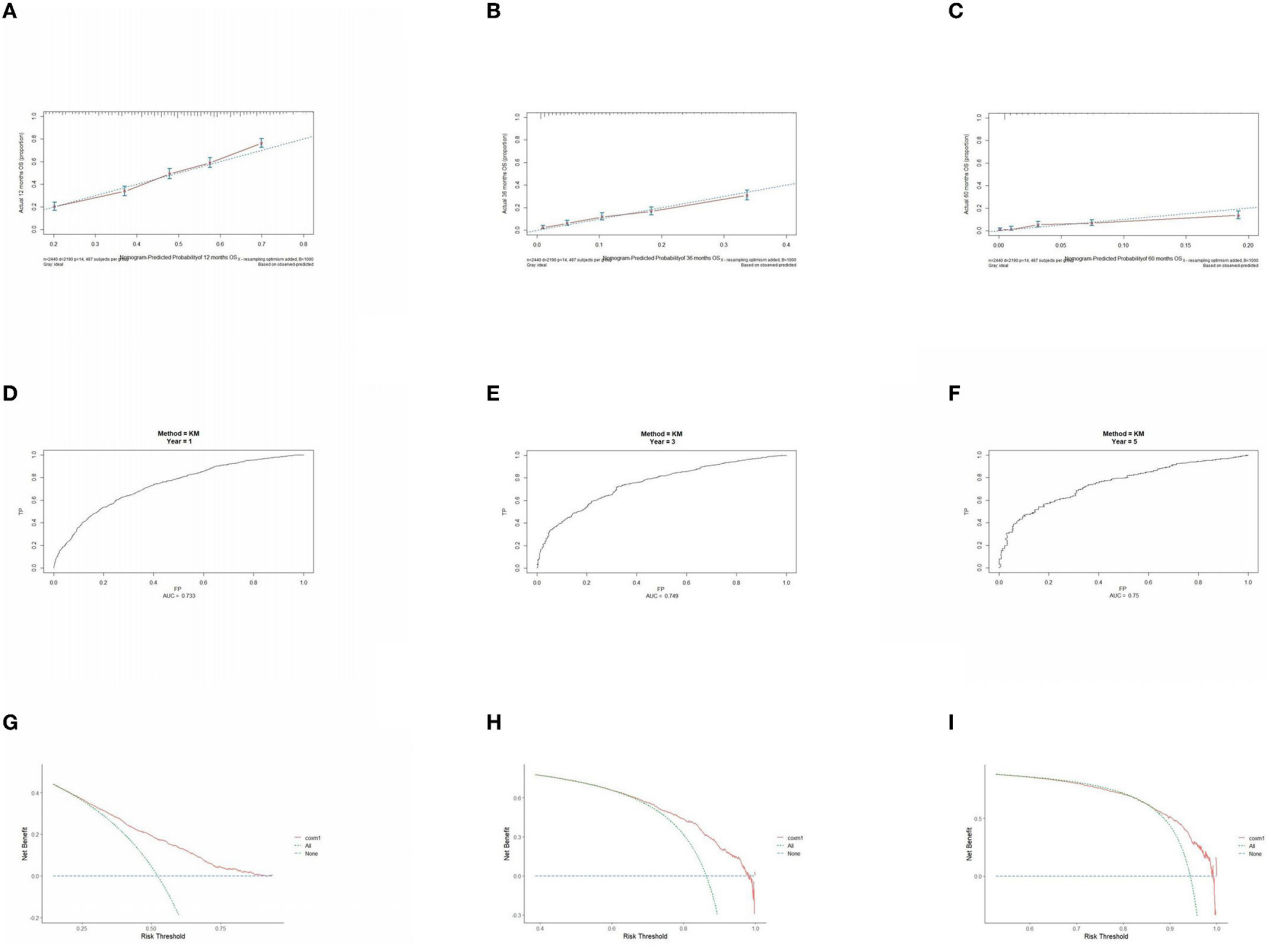
Performance verification of 1,3 and 5 year nomograms for stage IV in the training set. Nomograms 1,3 and 5 year calibration curves **(A–C)**, 1,3 and 5 year time dependent ROC curves **(D–F)**, 1,3 and 5 year decision curve analysis **(G–I)**.

**Figure 6:**
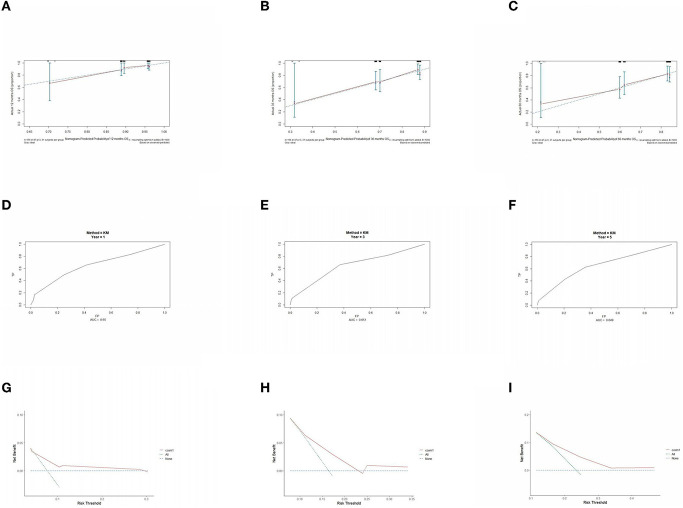
Performance verification of 1,3 and 5 year nomograms for stage I/II operable in the validation set. Nomograms 1,3 and 5 year calibration curves **(A–C)**, 1,3 and 5 year time dependent ROC curves **(D–F)**, 1,3 and 5 year decision curve analysis **(G–I)**.

**Figure 7:**
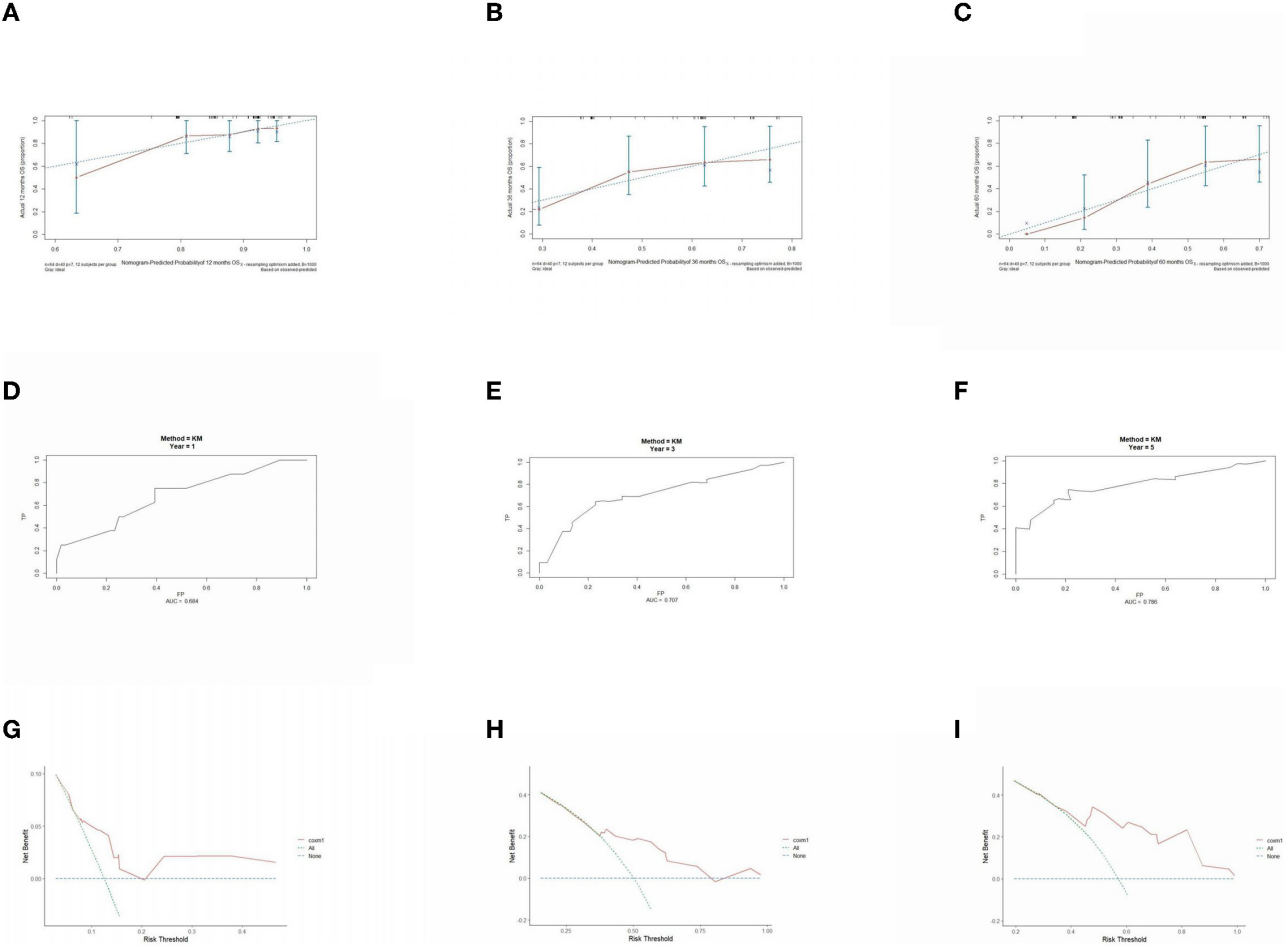
Performance verification of 1,3 and 5 year nomograms for stage III operable in the validation set. Nomograms 1,3 and 5 year calibration curves **(A–C)**, 1,3 and 5 year time dependent ROC curves **(D–F)**, 1,3 and 5 year decision curve analysis **(G–I)**.

**Figure 8:**
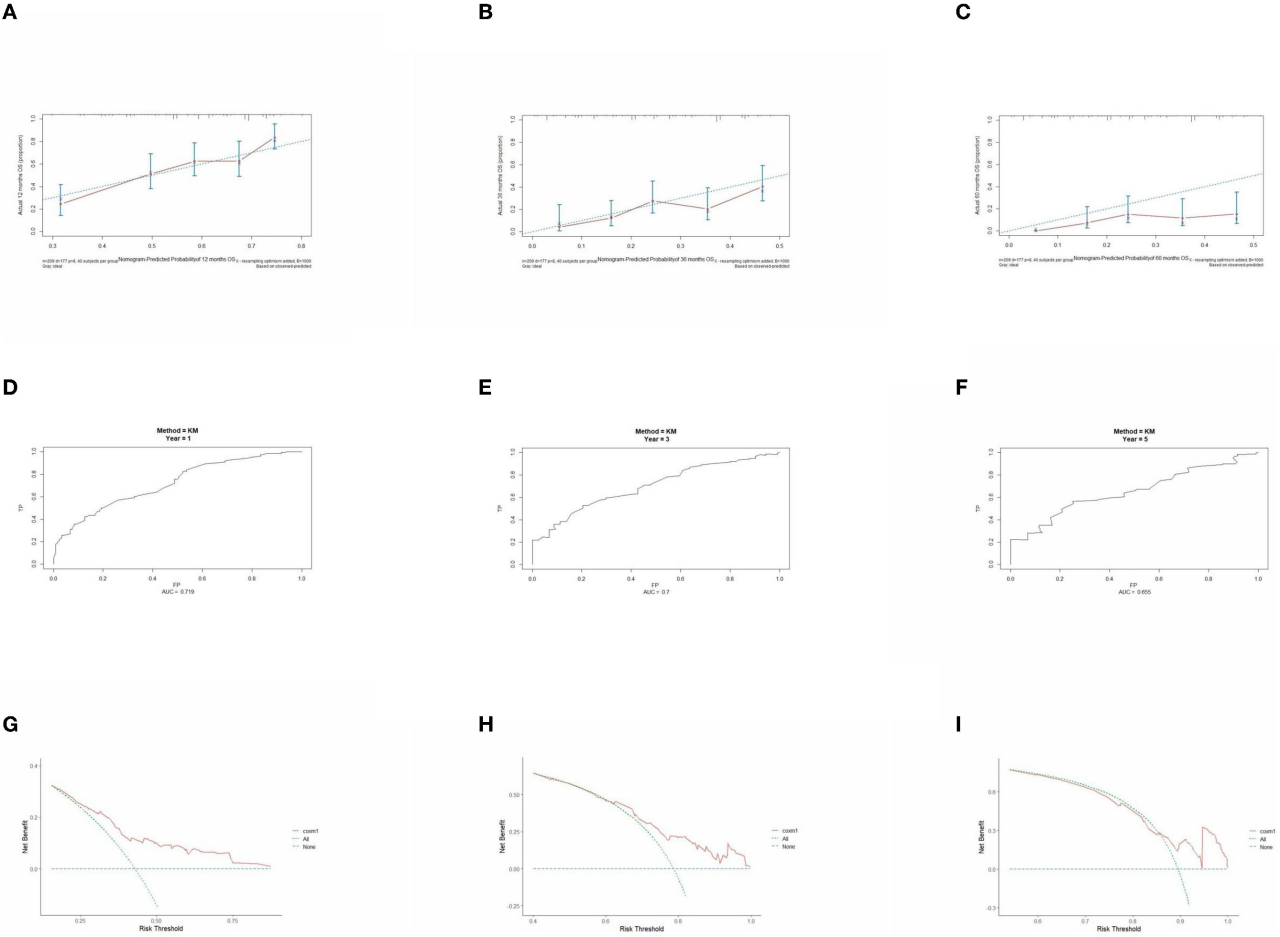
Performance verification of 1,3 and 5 year nomograms for stage III inoperable in the validation set. Nomograms 1,3 and 5 year calibration curves **(A–C)**, 1,3 and 5 year time dependent ROC curves **(D–F)**, 1,3 and 5 year decision curve analysis **(G–I)**.

**Figure 9:**
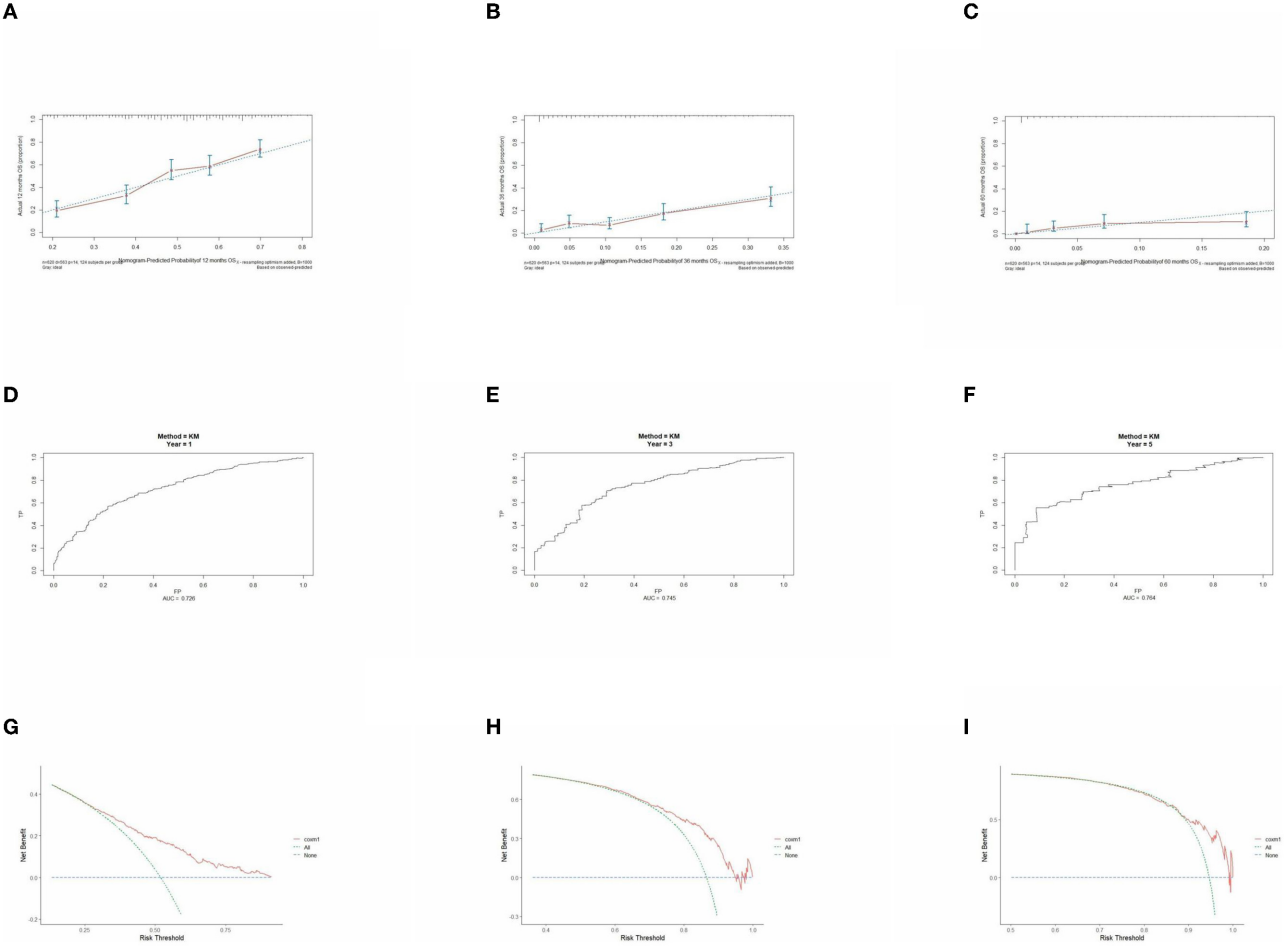
Performance verification of 1,3 and 5 year nomograms for stage IV in the validation set. Nomograms 1,3 and 5 year calibration curves **(A–C)**, 1,3 and 5 year time dependent ROC curves **(D–F)**, 1,3 and 5 year decision curve analysis **(G–I)**.

The prognostic model was validated using the training set data (Figures 2A–I–5A–I). In the stage I/II operable group (Figure 2), the calibration curves at 1, 3, and 5 years (Figures 2A–C) had good agreement, and the predicted probability was more consistent with the actual probability of occurrence. The time-dependent ROC (Figures 2D–F) curve and the calculated C-index values (0.657, 0.597, and 0.628) showed that the discrimination accuracy at 1, 3, and 5 years was poor, and in the clinical decision curve (Figures 2G–I), all suggested good clinical application value. In the stage III operable group (Figure 3), the calibration curve (Figures 3A–C) showed good agreement at 1, 3, and 5 years. The time-dependent ROC curve (Figures 3D–F) suggested good discrimination (0.753, 0.712, and 0.705), and the clinical decision curve (Figures 3G–I) also suggested good clinical application value. In the stage III inoperable group (Figure 4), the calibration curves (Figures 4D–F) were also in good agreement at 1, 3, and 5 years (0.697, 0.738, and 0.720). In the stage IV group (Figure 5), calibration curves (Figures 5A–C), time-dependent ROC curves (0.733, 0.749, and 0.750) (Figures 5D–F), and clinical decision curves (Figures 5G–I) showed good agreement, discrimination, and clinical utility. Internal validation was performed using the validation set (Figures 6A–I–9A–I), and the results were consistent with the training set.

### 3.6. K-M survival curve

**Figure 10:**
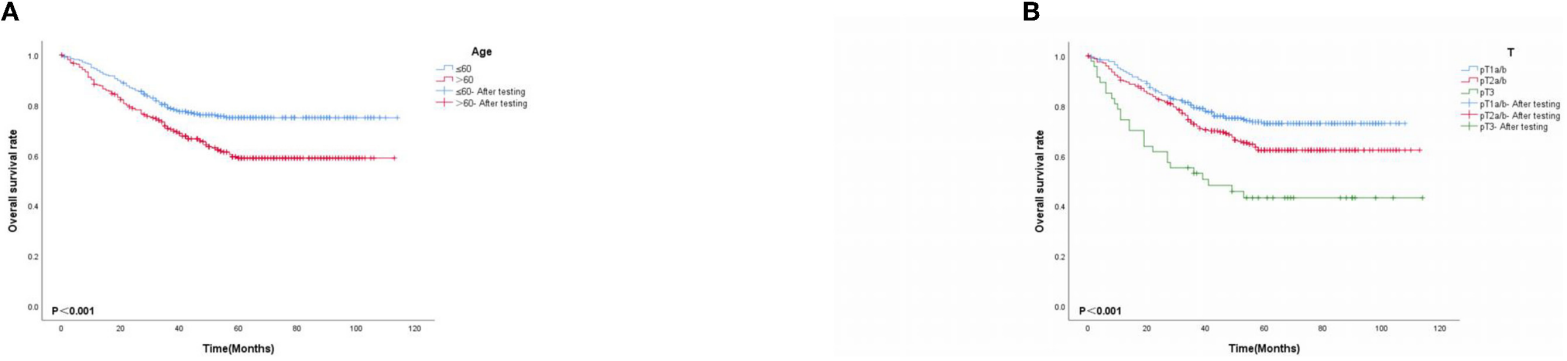
**(A, B)** K-M survival curves for significant indicators in stage I/II operable group.

**Figure 11:**
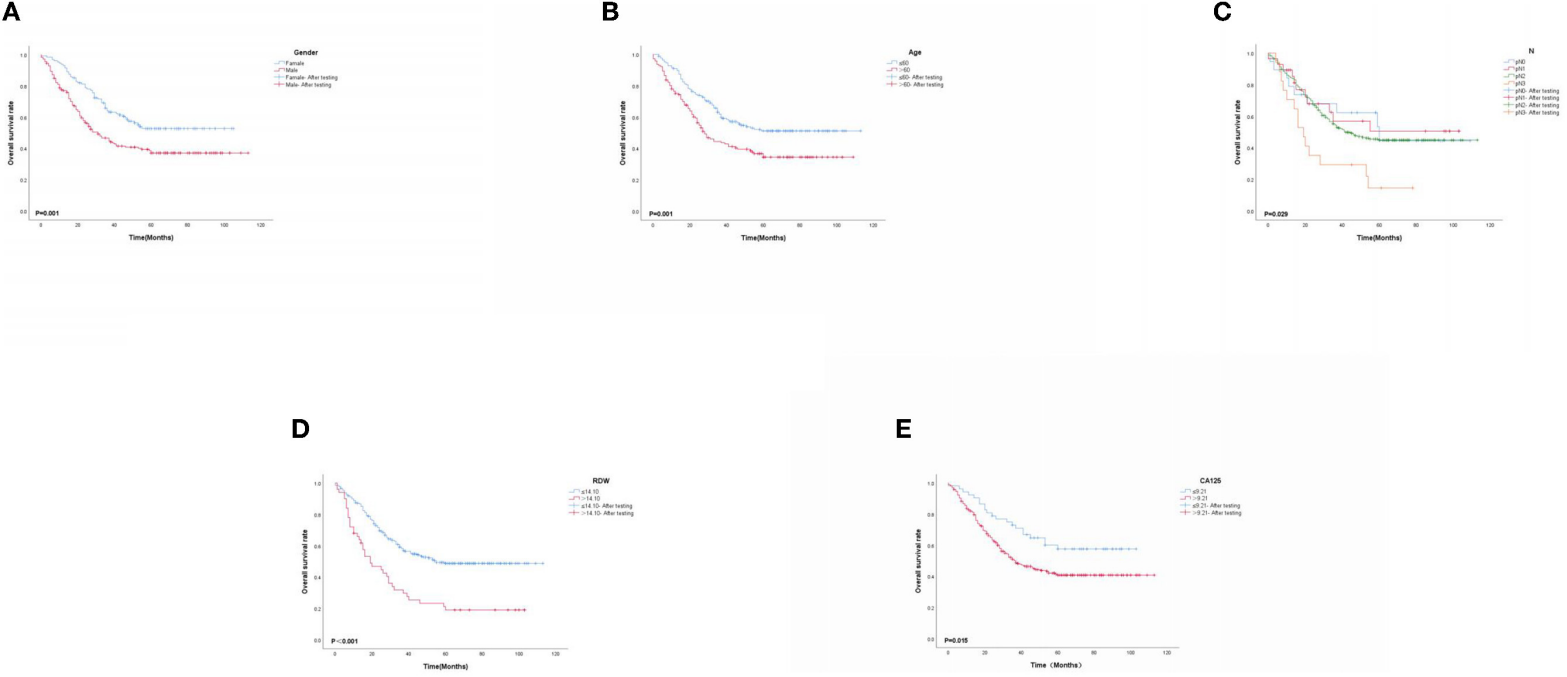
**(A–E)** K-M survival curves for significant indicators in stage III operable group.

**Figure 12:**
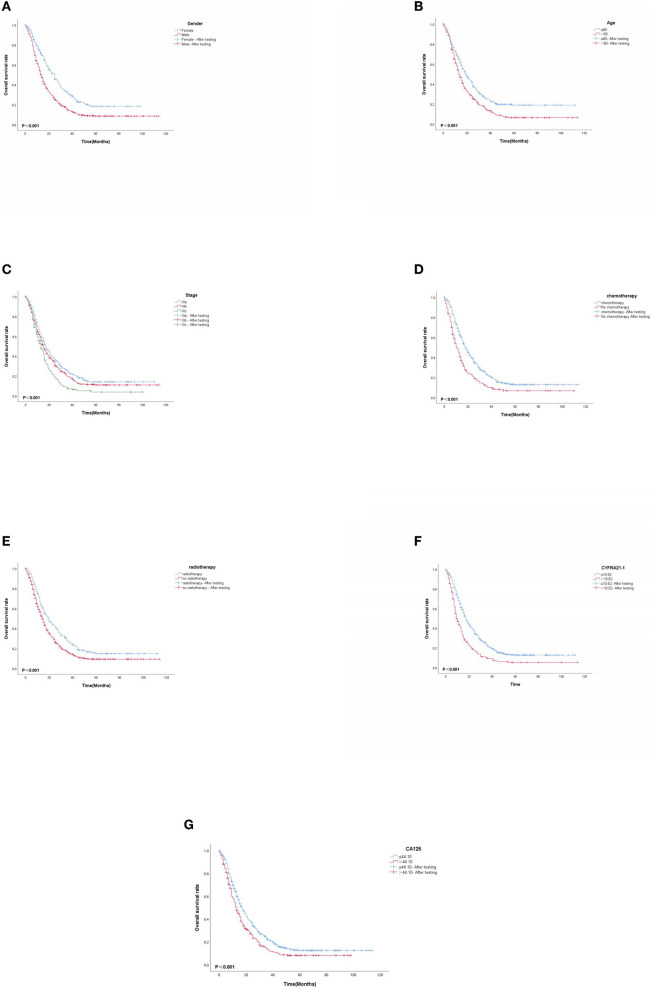
**(A–G)** K-M survival curves for significant indicators in stage III inoperable group.

**Figure 13:**
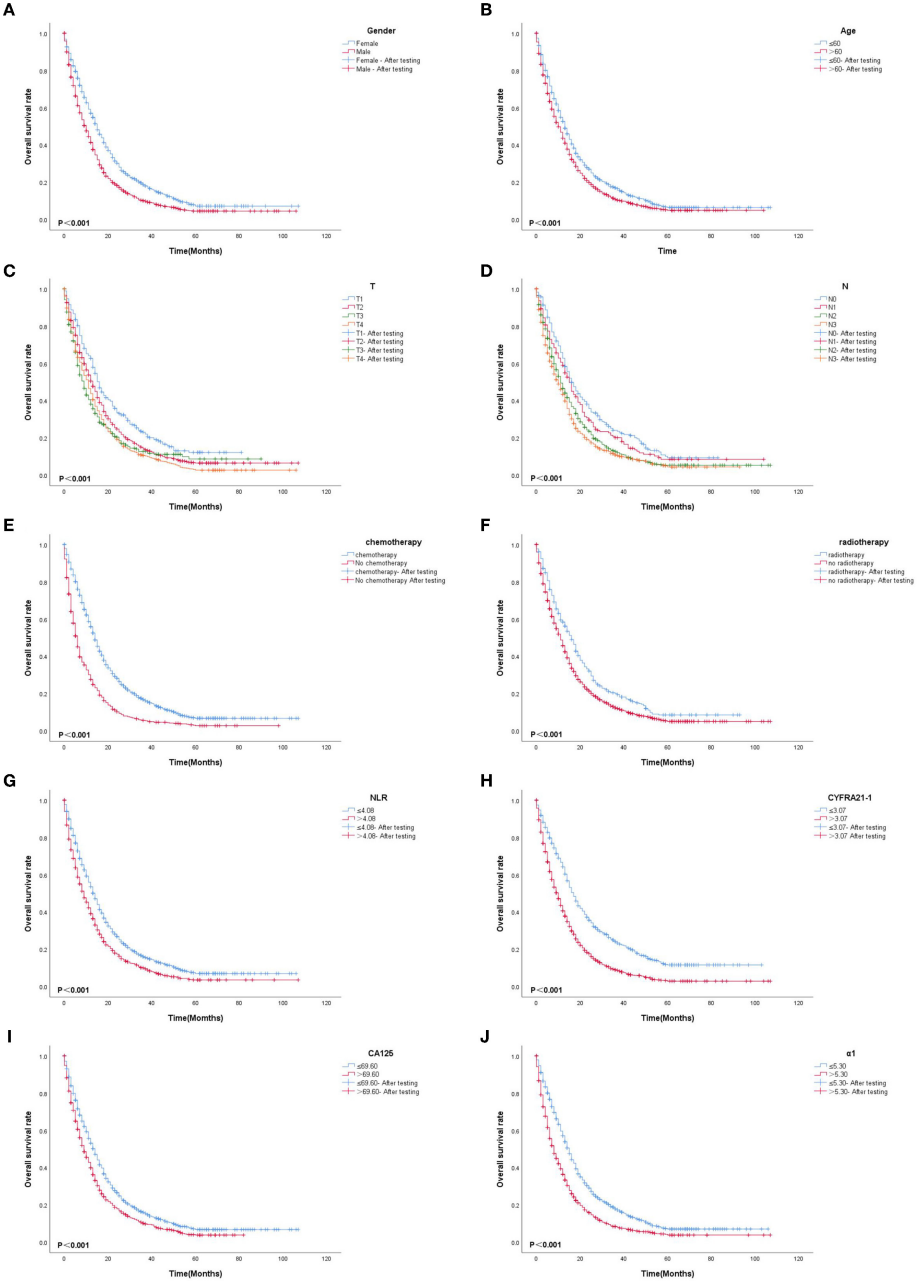
**(A–J)** K-M survival curves for significant indicators in stage IV group.

Based on multivariate Cox proportional-hazard regression analysis to screen independent risk factors, we established the K-M method to draw survival curves and used a log-rank test to assess survival differences. In the stage I/II operable group (Figures 10A, B), there was a significant difference in the overall production rate between the two groups by stratified log-rank test for age (*p* < 0.001) and T (*p* < 0.001). In the stage III operable group (Figures 11A–E), there was a significant difference in the overall production rate between the two groups by stratified log-rank test for gender (*p* = 0.001), age (*p* = 0.001), N (*p* = 0.029), RDW (*p* < 0.001), and CA125 (*p* = 0.015). In the stage III inoperable group (Figures 12A–G), there was a significant difference in the overall production rate between the two groups by stratified log-rank test for gender (*p* < 0.001), age (*p* < 0.001), stage (*p* < 0.001), chemotherapy (*p* < 0.001), radiotherapy (*p* < 0.001), CYFRA21-1 (*p* < 0.001), and CA125 (*p* < 0.001). In the stage IV group (Figures 13A–J), there was a significant difference in the overall production rate between the two groups by stratified log-rank test for gender (*p* < 0.001), age (*p* < 0.001), T (*p* < 0.001), N (*p* < 0.001), chemotherapy (*p* < 0.001), radiotherapy (*p* < 0.001), NLR (*p* < 0.001), CYFRA21-1 (*p* < 0.001), CA125 (*p* < 0.001), and α1-globulin (*p* < 0.001), and all *p* < 0.05.

## 4. Discussion

Lung cancer is a highly prevalent malignant tumor with the highest incidence and mortality worldwide, second only to breast cancer. Of all lung cancer types, NSCLC is 85% making it important to study NSCLC. Early lung cancer is mainly treated through surgery. According to relevant literature, the median OS time of patients with early NSCLC who undergo surgery is 7.9 years. When patients exhibit clinical manifestations such as cough, chest pain, and hemoptysis, they are already in the middle and advanced stages. Although the survival rate has significantly increased with individualized and precise treatment of lung cancer, the median OS time is just 4–34 months (18), which is substantially different from early lung cancer prognosis. Therefore, it is equally important to study the different stages of lung cancer.

According to related studies, the tumor microenvironment plays an essential role in the occurrence and development of malignant tumors. With more in-depth research, the relationship between inflammation and malignant tumors is being increasingly recognized. The inflammation mechanism promoting the growth of malignant tumors may be described as: inflammation releases cytokines and transcription factors are up-regulated, leading to the generation and accumulation of a large number of oxygen-free radicals, which cause DNA damage and breakage in parenchymal cells, including stem cells. The overexpression of proto-oncogenes, loss of tumor suppressor gene function, and up-regulation of genes promoting the cell cycle lead to abnormal cell proliferation, thereby promoting the occurrence of tumors.

The presence of NLR, LMR, NMR, PLR, PDW, RDW, and SII in blood cells as inflammatory indicators have been widely confirmed to predict the survival prognosis of malignant tumors effectively. Cytokines and inflammatory mediators produced by inflammatory cells could produce a series of related stress responses, trigger inflammatory cells and protein aggregation, and bring about the biological effect of oxidative cell damage. These activities interfere with the stability of the body's microenvironment, thereby accelerating tumor growth, invasion, metastasis, and other processes that affect the prognosis of tumors (19–22). These tumors include α1-antitrypsin (α1-AT), α1-acid glycoprotein (α1-AG), C-reactive globulin (CRP) (23), etc., in acute phase response proteins. Their increased levels could be used to predict the prognosis of malignant tumors.

However, there are few studies on the application value of acute phase response proteins in lung cancer. In protein electrophoresis, the vast majority of acute phase response proteins exist in the α1-globulin and α2-globulin, and an increase in α1-globulin and α2-globulin can fully reflect the inflammatory status (24). We innovatively included protein electrophoresis results to find an association between acute phase recognition reactive protein and lung cancer. Moreover, with malignant tumors being consumptive diseases, their nutritional indicators, such as Alb and PNI, are also considered to affect their prognosis. Recent research on tumor markers has found that they can assist in the diagnosis and have a certain value for survival prognosis, treatment response, recurrence, and metastasis (25). Therefore, it is of great significance to include inflammation, nutritional indicators, and tumor markers as influencing factors and conduct a large-sample retrospective study.

The current X-tile software is commonly used to intercept optimal cut-off values for survival analysis. In this study, we intercepted the optimal cut-off value for quantitative data according to different stages and divided it into two categories. Using the training set for univariate and multivariate Cox proportional-hazard regression, we found that only age and T were independent risk factors in the group receiving surgery in stage I/II, which may be associated with chronic inflammation caused by malignant tumors that could not stimulate the body for a long time because of the short onset time. At the same time, adjuvant chemotherapy performed after surgery was not an independent risk factor affecting the prognosis. This was compatible with Xue et al. (26) view that receiving surgery was still the most significant influencing factor for early lung cancer. In the stage III operable group, gender, age, N, RDW, and CA125 were independent risk factors. According to Qi-Fan et al. (18), with the progression of the disease, T increased, and N had a greater effect on prognosis than the T stage. Similarly, we found that whether chemotherapy and radiotherapy were performed after surgery was not an influencing factor. However, in this study, because fewer patients underwent surgery in stage III, there were more bias factors, and the statistical power was weakened, which required further verification.

In the stage III inoperable group, gender, age, stage, radiotherapy, chemotherapy, CYFRA21-1, and CA125 were independent risk factors. For stage III inoperable patients, whether they were treated, including chemotherapy and radiotherapy, were strong influencing factors. Zhi and Jun (27) also found that in advanced lung cancer, tumor markers could predict the prognosis more significantly than in the early stage. Most lung cancers were diagnosed at an advanced stage and missed the timing of surgery, so active and effective treatment was particularly important, with the greatest impact of chemotherapy. As described by clinical and demographic characteristics, we believe that chronic inflammatory stimulation for a long time leads to higher and lower inflammatory markers, tumor markers, and nutritional indicators in patients. The large sample data showed that gender, age, T, N, chemotherapy, radiotherapy, NLR, CYFRA21-1, CA125, and α1-globulin were independent risk factors in the stage IV group. Nutritional indicators were not significant in any of the staged risk strata, which may be associated with little difference in nutritional status per patient in each stratum, but nutritional indicators were different between groups and require further study.

While undertaking risk stratification according to stage, we also innovatively combined inflammation, nutritional indicators, and tumor markers to predict prognosis in NSCLC. To the best of our knowledge, this is the first time it has been attempted. Though this study had a large sample of data, it has limitations. With the small sample size for early lung cancer, bias factors may limit statistical power. Because of the retrospective nature of the data collection and the failure to include some known prognostic parameters such as tumor cell differentiation, vascular invasion, and perineural invasion, and some important molecular factors (such as EGFR mutation, ALKEML4 fusion), more rigorous prospective studies are needed to validate, and further efforts are required to improve this model in terms of wider geographic recruitment and integration of some other factors.

## Data availability statement

The original contributions presented in the study are included in the article/supplementary material, further inquiries can be directed to the corresponding author.

## Ethics statement

Ethical review and approval was not required for the study on human participants in accordance with the local legislation and institutional requirements. Written informed consent from the patients/participants or patients/participants' legal guardian/next of kin was not required to participate in this study in accordance with the national legislation and the institutional requirements.

## Author contributions

BX, QZ, and SH had full access to all the data in the study and take responsibility for the integrity of the data and the accuracy of the data analysis. Concept, design, and statistical analysis: XL, BX, QZ, and SH. Acquisition, analysis, and interpretation of data: XL. Drafting of the manuscript: XL, BX, and YF. Funding: XL and YF. Administrative: XL, BX, QZ, SH, and YF. Study supervision: XL, QZ, and YF. All authors contributed to the article and approved the submitted version.
